# Flavonoid Synthesis and Metabolism During the Fruit Development in Hickory (*Carya cathayensis*)

**DOI:** 10.3389/fpls.2022.896421

**Published:** 2022-05-09

**Authors:** Jia-Hui Chen, Na Hou, Xv Xv, Da Zhang, Tong-Qiang Fan, Qi-Xiang Zhang, You-Jun Huang

**Affiliations:** ^1^Zhejiang Provincial Key Laboratory of Forest Aromatic Plants-based Healthcare Functions, Zhejiang A&F University, Hangzhou, China; ^2^Guizhou Academy of Forestry, Guiyang, China

**Keywords:** *Carya cathayensis*, hickory, flavonoid, fruit development, transcriptome, metabonomics

## Abstract

Hickory (*Carya cathayensis*) kernel is rich in powerful bioactive flavonoids, which can remove excess free radicals in the human body and play an important role in regulating the physiological metabolism of the plant. This study investigated the changes of flavonoids in hickory exocarp and embryo during development. In this study, 72 DEGs involved in the regulation of flavonoid biosynthesis in fruits were identified, and *TT4*, *CCoAOMT1*, *UGT71D1*, *C4H*, *F3H*, *TT8*, *FLS1*, and *LDOX* were identified as the core genes of flavonoid biosynthesis. A total of 144 flavonoid-related metabolites were detected by metabolite analysis. Transcriptome and metabolome analysis combined to construct the flavonoid biosynthesis regulatory pathway in the development stage of hickory fruit. Our results provide a theoretical basis for the exploration and regulation of functional genes related to flavonoid biosynthesis and metabolism in hickory and other plants and the breeding of new walnut varieties.

## Introduction

Hickory (*Carya cathayensis*) is an important and widely planted nut tree species in Zhejiang and Anhui provinces of China. Because of its high nutritional value and unique flavor, Hickory has become more and more popular in southern China in recent years. Flavonoids produced by plants are important dietary components in animal systems. Flavonoids and phenolic compounds have a wide range of properties, including antibacterial, antifungal, antiviral, and anticancer activities (Soto-Vaca et al., 2012; Khodadadi et al., 2016). Many flavonoids play roles as templates in the development of new drugs (Wright et al., 2013). Hickory and walnut are rich sources of phytochemicals such as flavonoids and phenolic compounds (Khodadadi et al., 2020), containing large amounts of healthy monounsaturated fatty acids, high antioxidants, and a range of phytochemicals (Bolling et al., 2011). The main components of hickory fruit are phenolic acids, flavonoids, and naphthoquinones. Nuts are also a rich source of dietary fiber, protein, minerals, and B vitamins, especially thiamine (Zhu et al., 2008). Recent studies have highlighted that the health benefits of healthy consumption of these nuts are associated with reduced incidence of various diseases, such as tumors, edema, hyperglycemia, and hyperlipidemia (Hilbig et al., 2018).

Flavonoids are natural products belonging to general polyphenols, which are generally considered essential for the survival of plants (Quideau et al., 2011). They are the most abundant secondary metabolites in plants, with more than 10,000 structural variations (Quideau et al., 2011). Their synthesis contributes to plant protection and signal transmission (Delaux et al., 2012), can resist pathogens, and protect plants from ultraviolet radiation. Flavonoids can also stimulate various activities of microorganisms, plants, and animals (Buer et al., 2010), regulate the level of reactive oxygen species (ROS) in plant tissues, and provide pigment for various tissues, including flowers (Peer and Murphy, 2007; Agati et al., 2012; Davies et al., 2012). In addition, they are necessary for the signal transduction of legume rhizobia symbiosis and play an important role in root and stem development (Zhang et al., 2009). As an antioxidant, flavonoids can reduce ROS production (Mierziak et al., 2014).

Studies on the biosynthesis and metabolism of flavonoids in fruit trees have been carried out in grape, kiwi, citrus, and other trees. Existing research results provide a theoretical basis for the biosynthesis pathway of plant flavonoids but mainly focus on specific influencing factors (temperature, light, stress, etc.), key enzyme (PAL, CHI, etc.) genes, and transcription factors (MYB, BHLH, WD40, BZIP, etc.) in a single direction.

The studies on the metabolic pathway of flavonoids and related enzymes, genes, and regulatory factors in hickory are still unclear. This study used the transcriptome and metabolome sequencing techniques to analyze the gene expression profiles and metabolites in the exocarp and embryo of hickory at different stages after flowering and pollination. The dynamic changes of gene transcription in the process of flavonoid synthesis in hickory were analyzed in-depth, and the key enzymes, genes, and transcription factors of the flavonoid synthesis pathway in hickory were explored to provide more information for the study of flavonoid synthesis in hickory and other plants and the mining of related functional genes. At the same time, it laid a theoretical foundation for the research on the flavonoid proteome and translation group and the breeding of hickory varieties.

## Materials and Methods

### Plant Materials

The trials were carried out in 2016 and 2020. In 2016, the material was collected from a hickory tree (*C. cathayensis*) in the fruit garden (N30°15′18. 1″, E119°43′43″) of Donghu Campus of Zhejiang A & F University, which was a 17-year-old tree with the whole-genome sequencing strain (ZAFU-1). After pollination, 100 fruits were randomly picked from east, south, west and north of the middle and upper part of the tree crown at 85d (DAP85, middle cotyledon), 91d (DAP91, late cotyledon), and 127d (DAP127, fruit ripening stage), respectively. The samples were frozen quickly in liquid nitrogen and stored in a refrigerator at −80°C after sampling (separation of exocarp and embryo). In 2020, three test trees were planted at 5 m spacing in Zhi’nan Village (N30°22′48″, E119°34′48″), Taihuyuan Town, Lin’an District, Hangzhou City, Zhejiang Province. Three trees are approximately 30 years old, with similar tree potential and a growing environment. One hundred fruits at each stage and each tree were randomly picked from the east, south, west, and north of the middle and upper part of the tree crown at 85d (DAP85, middle cotyledon stage), 100d (DAP100, late cotyledon stage), 109d (DAP109, complete cotyledon stage), and 127d (DAP127, fruit ripening stage) after pollination. The samples were frozen quickly in liquid nitrogen and stored in a refrigerator at −80°C after sampling (separation of exocarp and embryo).

### Methods

#### Transcriptome Data Analysis

Total RNA was extracted by the kit method, and a library was established. Qubit2.0 fluorescence analyzer was used for preliminary quantitative analysis, and the library was diluted to 1.5 ng/ul. Agilent 2,100 BioAnalyzer was used to detect the insert size of the library to ensure the quality of the library. The Illumina PE150 sequencing generates pairwise end readings of 150 bp.

The raw data are filtered to remove reads with adapters, reads with N (N denoting unascertainable base information), and reads with low quality (reads with Qphred ≤20 bases accounting for more than 50% of the entire read length) were removed. The content of Q20, Q30, and GC of clean data was calculated. The reference genome and gene model annotation files were directly downloaded from the Chinese hickory genome website.^1^ HISAT2 (Pertea et al., 2015) v2.0.5 was used to construct the reference genome index and compare the clean reads at the paired ends against the reference genome. New gene prediction was performed using String Tie (Kim et al., 2019; 1.3.3b). Feature Counts (Liao et al., 2014; 1.5.0-P3) were used to calculate the reading mapped to each gene. The FPKM of each gene was calculated according to the length of the gene. The FPKM data were analyzed by DESeq2 (Love et al., 2014) software (1.20.0) for differential expression analysis between the two combinations. Benjamini and Hochberg’s method was used to adjust the value of p to control the error detection rate. The adjusted *p* < 0.05 genes were retained as differentially expressed genes (DEGs). Clustering the expression patterns of all the different genes was carried out, and then, the gene sets conforming to certain biological characteristics were selected for trend analysis. Cluster Profiler (Yu et al., 2012; 3.4.4) software was used for GO enrichment analysis of DEGs, and the value of p was calculated by hypothesis test and corrected by FDR. The Go term and Pathway significantly enriched with Qvalue≤0. 05 as the threshold. The DEGs of flavonoid metabolism were obtained. GSEA analysis was performed for GO and KEGG data sets using a local version of the GSEA tool.^2^

PPI analysis of DEGs was based on the STRING database, using target genes to construct a network. Otherwise, Diamond (Buchfink et al., 2015; 0.9.13) was used to align the target gene sequence with the selected reference protein sequence. Then, the network was established based on the known interactions of the selected reference species. The software rMATS (Shen et al., 2014; 3.2.5) analyzed the alternative splicing events, which mainly included SE, RI, MXE, A5SS, and A3SS. GATK (McKenna et al., 2010; 3.7) software was used to analyze the various sites of the sample data, and SNPEFF (Cingolani et al., 2012; 4.3q) software was used to annotate the variation sites. To construct the WGCNA network, the expression correlation coefficient among genes was firstly calculated. Then, the POWER value, which made the data conform to the scale-free distribution, was found to construct the gene cluster tree. And the gene modules were divided according to the clustering relationship among genes. Correlation analyses between modules and module genes were performed, respectively, and the corresponding heat maps were drawn. Finally, the modules significantly related to a specific sample were selected.

GO/KEGG was performed for each gene module. In addition, the gene regulatory network map obtained Hub genes, which are at a pivotal position in the regulatory network of flavonoid metabolism in the exocarp and embryo.

#### Metabolome Data Analysis Process

Target-like metabolomics studies were conducted using liquid mass spectrometry (LC-MS) based on the highly sensitive SCIEX QTRAP^®^ 6,500+ mass spectrometry platform. The mass spectrometry detection process was divided into Blank sample (BLANK), IS standard mixed sample (IS mixed standard), quality control sample (QC), and experimental sample on computer detection. The SCIEXOSV1.4 software was used to open the offline mass spectrometry file (.wiff), and the integration and calibration of chromatographic peaks were carried out. The quality control of the QC sample data ensured the accuracy and reliability of the data results.

Then, the metabolites were analyzed by multivariate statistical analysis: hierarchical clustering (HCA), correlation analysis of metabolites, and functional analysis of metabolic pathways. The experimental samples were detected and qualitatively analyzed, and the triple quadrupole MRM model quantitatively analyzed the compounds. Pathway annotation and classification of identified metabolites were performed. Major databases such as KEGG, HMDB, and lipid map were used to label the identified metabolites. Correlation analysis of different metabolites was conducted. A statistical significance test was conducted for correlation analysis of metabolites, and the threshold was set as a value of *p* < 0. 05. Z-score analysis was performed to measure the relative quantitative value of metabolites on the same level. Finally, KEGG enrichment analysis of differential metabolites was conducted.

## Results

### Expression Analysis of Developmental Genes in Hickory Fruit

#### Gene Expression Profile Analysis in 2016

**Figure 1:**
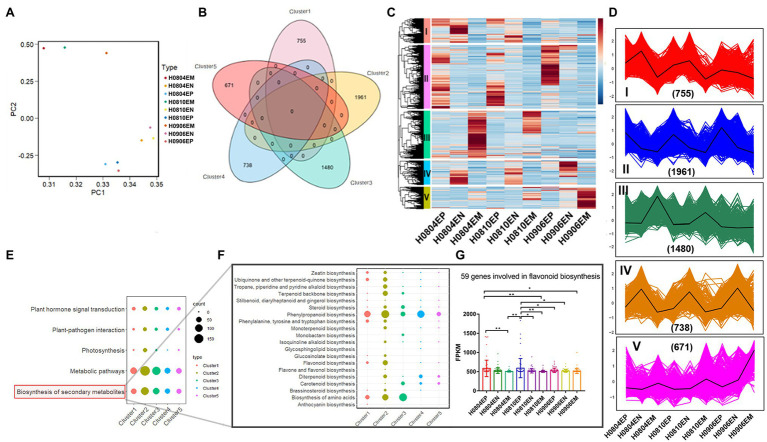
Differential expression and trends of genes related to fruit development in hickory and functional annotation of DEGs involved in flavonoid metabolism. **(A)** Inter-sample principal component analysis; **(B)** *K*-means clustering analysis; **(C)** Heat map of DEGs; **(D)** Differential gene trend analysis; **(E)** Top5KEGG pathway of differential genes; **(F)** Pathways related to secondary metabolite synthesis; and **(G)** Difference analysis of flavonoid synthesis genes.

Hickory exocarp and embryo were healthy developing at different development time points (Supplementary Figure S1). After log2 normalization, PCA analysis of the gene FPKM showed that principal components 1 and 2 contained 51.2 and 24.5% differences, respectively (Figure 1A). *K*-means clustering method was used to determine the optimal clustering *k* value of 5 (Figure 1B), and the difference analysis showed that a total of 5,605 genes were differentially expressed (Figure 1C). The gene trend analysis revealed the trends of 5 species at different developmental stages: I, II, III, IV, and V (Figure 1D).

#### Screening of Flavonoid Metabolism-Related Genes in 2016

To systematically analyze the metabolic pathways involved in the development of hickory fruit, KEGG functional enrichment analysis was performed on the differential transcripts of the above five trends. The results showed 485 differential genes enriched into 34 KEGG pathways. The top five include plant hormone signal transduction, plant-pathogen interaction, photosynthesis, metabolic pathways, and biosynthesis of Secondary metabolites (Figures 1E–G). KEGG pathway annotation results showed Phenylpropanoid biosynthesis, Phenylalanine metabolism, Flavonoid biosynthesis, Isoflavone biosynthesis, Flavone and flavonol biosynthesis, and Anthocyanin biosynthesis are related to the metabolism of flavonoids in hickory. A total of 37 genes were noted to be directly involved in flavonoid metabolism in the above pathways, which regulated the synthesis of related enzymes in the process of flavonoid metabolism in hickory (Figures 1E–G; Supplementary Table S1). In view of the importance of flavonoids to human health, further research will be focused on the synthesis of flavonoids during the development of hickory nuts in 2020.

#### Gene Expression Profile Sequencing and Comparative Statistics in 2020

Transcriptome sequencing was performed on 24 samples from the exocarp and embryos of four stages (DAP85, DAP100, DAP109, and DAP127), with three biological replicates. The clean reads obtained from each library accounted for more than 90% of the total reads. Approximately 95% ~ 97% of clean reads in each expression profile data could be mapped to the reference genome (Supplementary Table S2). And more than 80% of reads were mapped to the exon region (Supplementary Figure S2). The results showed that the mapped reads could be directly used to calculate the gene expression between different samples. The expression abundance of the 24 samples was concentrated between 0 and 0.5, and the expression abundance of the three biological replicates in the same developmental period was highly consistent. In contrast, the number and abundance of expressed genes in different developmental periods differed. The sequencing results could be used to analyze differential gene expression (Supplementary Figure S3).

### Prediction of New Transcript

**Figure 2:**
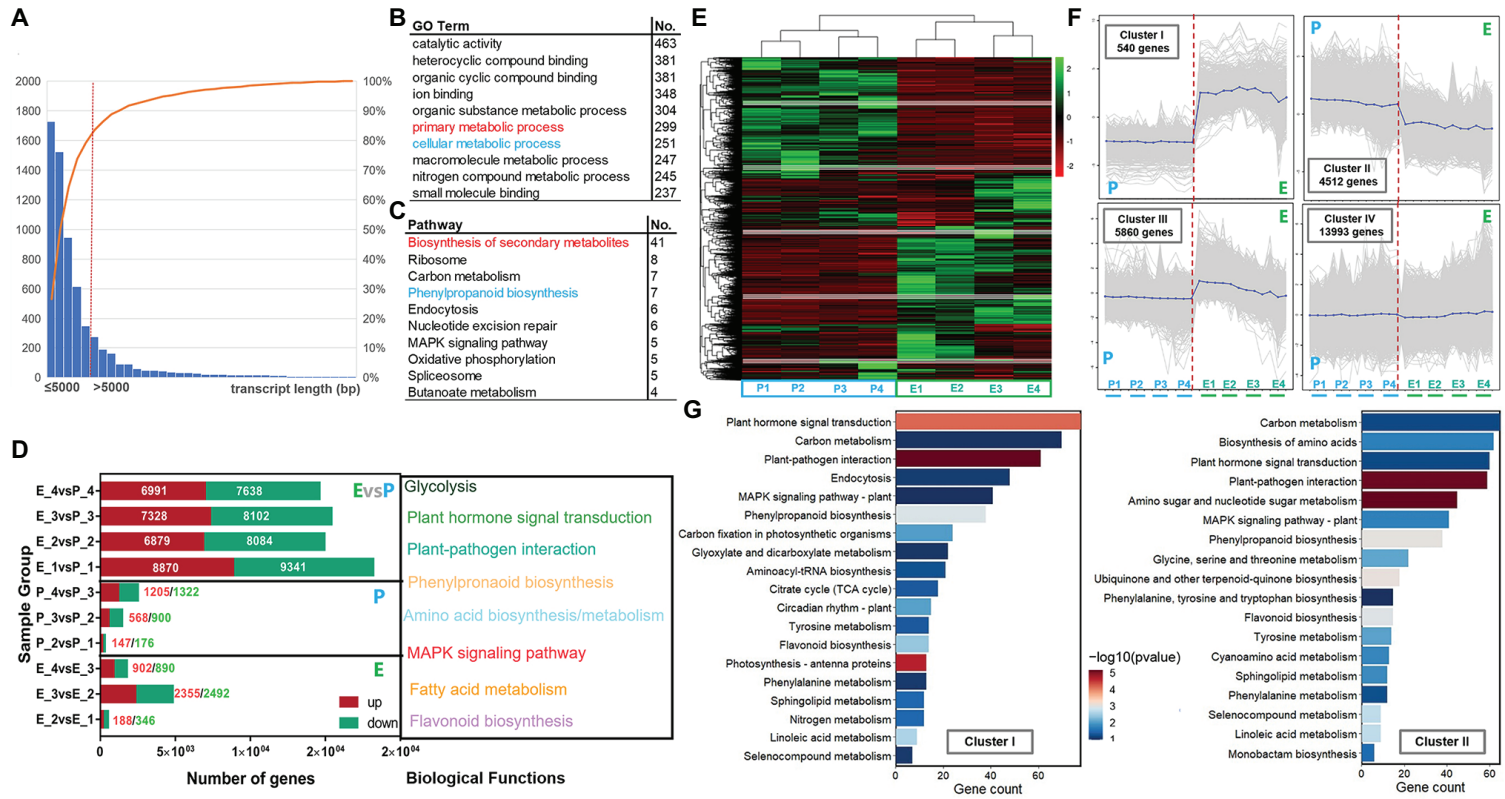
Functional annotation of new transcripts, expression of DEGs in exocarp and embryo at different developmental stages, trend analysis of DEGs, and KEGG enrichment analysis in hickory fruits. **(A)** Counts in transcripts; **(B)** GO terms of transcripts; **(C)** KEGG pathways of transcripts; **(D)** The expression of differential genes in the epicarp and embryo; **(E)** Differential expressed genes analysis during hickory fruit development; **(F)** Clusters of DEGs; and **(G)** KEGG pathway enrichment analysis of trend genes.

Stringtie assembled 3,505 transcripts, and more than 90% of the transcripts were less than 5 KB in length. GO and KEGG were used to annotate the functions of these transcripts. The results showed that 966 transcripts were mapped to different GO functional nodes. The top 10 GO terms were catalytic activity, heterocyclic compound binding, organic cyclic compound binding, ion binding, organic substance metabolic process, primary metabolic process, cellular metabolic process, macromolecule metabolic process, nitrogen compound metabolic process, and small molecule binding. KEGG pathway enrichment showed that 125 transcripts were enriched into 72 pathways. The top 10 pathways were Biosynthesis of secondary metabolites, Ribosome, Carbon metabolism, Phenylpropanoid biosynthesis, Endocytosis, Nucleotide excision repair, MAPK signaling pathway, Oxidative phosphorylation, and Spliceosome and Butanoate metabolism (Figures 2A–C).

### Expression Patterns of Differential Transcripts

PCA showed that the exocarp and embryos among different samples during the same development period were clustered in compact groups, indicating good repeatability. However, between the exocarp and embryo, and between the exocarp and embryo were separated at different development stages, showing a good specificity (Supplementary Figure S4A). The correlation between samples was close to 1, indicating high reproducibility and good gene expression heterogeneity in the exocarp and embryo at the same development stage (Supplementary Figure S4B). The result showed that the expression profile is qualified for differential gene expression analysis.

According to the sample relationship analysis result, DEGs were further screened under the condition of filter FDR < 0.05 and | log_2_FC | > 1. The expression of DEGs in various combinations is shown in Figure 2D. The number of DEGs in each combination was different, the maximum was 9,341, and the minimum was 147. The number of differentially upregulated genes in exocarp was 147 (P2 vs. P1), 568 (P3 vs. P2), and 1,205 (P4 vs. P3) at four developmental stages, respectively. The number of downregulated differential genes was 176 (P2 vs. P1), 900 (P3 vs. P2), and 1,322 (P4 vs. P3). The number of differentially upregulated genes in the embryo was 188 (E2 vs. E1), 2,355 (E3 vs. E2), and 902 (E4 vs. E3) at four stages, respectively. The number of downregulated differential genes was 346 (E2 vs. E1), 2,492 (E3 vs. E2), and 890 (E4 vs. E3), respectively. In the same development period, the number of differentially upregulated genes was 8,870 (E1 vs. P1), 6,879 (E2 vs. P2), 7,328 (E3 vs. P3), and 6,991 (E4 vs. P4), respectively. The number of downregulated genes was 9,341 (E1 vs. P1), 8,084 (E2 vs. P2), 8,102 (E3 vs. P3), and 7,638 (E4 vs. P4), respectively. Notably, the DEGs number in different parts of the same developmental stage was more than that of DEGs in different developmental stages of the same part. The number of downregulated differential genes was always higher than that of upregulated genes and fruit development. These DEGs mainly involved Glycolysis, Plant hormone signal transduction, Phenylpropanoid biosynthesis, Amino acid biosynthesis/metabolism, MAPK signal pathway, Fatty acid metabolism, and Flavonoid biosynthesis.

### Differential Gene Analysis of Flavonoid Biosynthesis During the Development of Hickory Fruit

#### Expression Trend Analysis of Differential Genes

Four gene expression patterns were generated through clustering (Figure 2E) and trend analysis of DEGs in the exocarp and embryo of the four development stages, denoting Cluster I-IV (Figure 2F). GO/KEGG functional enrichment analysis showed that Cluster I and Cluster II became two dominant enrichment trends in the exocarp and embryo at different developmental stages. The expression level of genes in Cluster I was significantly higher in the embryo than in the exocarp. In contrast, the expression level of genes in ClusterII was higher in the exocarp than in the embryo. Furthermore, the expression level of genes in ClusterII decreased gradually with the fruit development. The GO and KEGG enrichment pathway analysis showed that genes in the two significant trends of Cluster I-II were mainly involved in primary metabolism, such as carbon metabolism and fatty acid biosynthesis, and secondary metabolism, such as phenylpropane metabolism and flavonoid synthesis. These results indicated that the metabolism of flavonoids was involved in the exocarp and embryo during the fruit development.

#### Differential Gene Analysis of Flavonoid Synthesis

**Figure 3:**
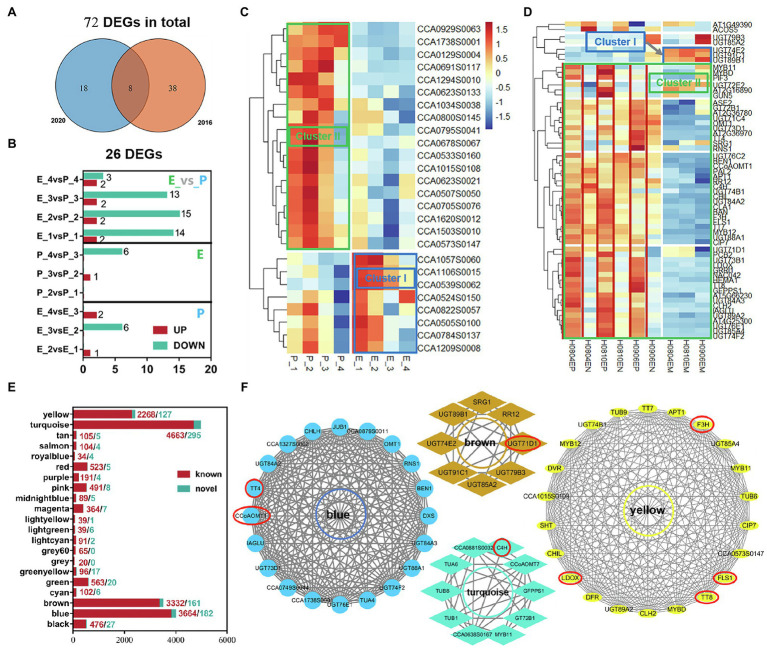
Differential genes involved in flavonoid biosynthesis, module gene division and KEGG functional enrichment analysis, and gene network regulation in flavonoid biosynthesis module. **(A)** Venn plots of transcriptome data in 2020 and 2016; **(B)** The number of different genes involved in flavonoid synthesis between groups; **(C,D)** Heat map of DEGs in 2016 and 2020; **(E)** Module gene statistics; **(F)** The genes in the red circle are the core genes in each module. In **(C,D)**, the blue frame is the gene set in the cluster I trend, and the green frame is the gene set in the Cluster II trend. Genes marked in red are sets of genes highly expressed in the exocarp.

KEGG functional enrichment analysis was performed on the two trends of ClusterI-II with significant differential genes in exocarp and embryo at four developmental stages of hickory to obtain the related pathways of flavonoid synthesis during fruit development (Figure 2G). First, the genes involved in flavonoid biosynthesis were screened out from Cluster I-II. Then, through joint analysis of transcriptome data in 2016 and 2020, a total of 72 (46 in 2016 and 26 in 2020) DEGs involved in flavonoid biosynthesis were uncovered from the transcriptome data (Figure 3A). These DEGs were mainly between tissues rather than within tissues, and most of them were downregulated (Figure 3B). Through the analysis of the expression levels of these DEGs, it was found that the expression levels of most of the DEGs decreased gradually with the development process, such as CCA1294S0010, CCA0623S0133, and CCA0795S0041, which were specifically highly expressed in the exocarp. At the same time, a class of highly expressed differential genes, such as CCA1057S0060, CCA1106S0015, and CCA0539S0062, were found to decrease expression levels gradually with fruit development (Figure 3C). These results indicated that flavonoid metabolism mainly occurred in the middle cotyledon stage (DAP85) and late cotyledon stage (DAP100) during fruit development. The flavonoid metabolism was highly active in inner and exocarp.

Seventy-two DEGs were further mapped to two expression trends (Cluster I-II; Figures 3C,D; Supplementary Table S3). Of them, 12 DEGs related to flavonoid biosynthesis were significantly enriched in Cluster I, including *C4H*, *BAN*, and *CCoAOMT7*. And 60 DEGs related to flavonoid biosynthesis were significantly enriched in Cluster II, including *F3H*, *TT4/7/8*, *CCoAOMT1*, *LDOX*, and *BEN1*. Hence, the activity of flavonoid synthesis in exocarp was significantly stronger than that in the embryo.

### Hub Genes for Flavonoid Biosynthesis During the Fruit Development

#### Construction of Flavonoid Co-expression Module During Fruit Development

**Figure 4:**
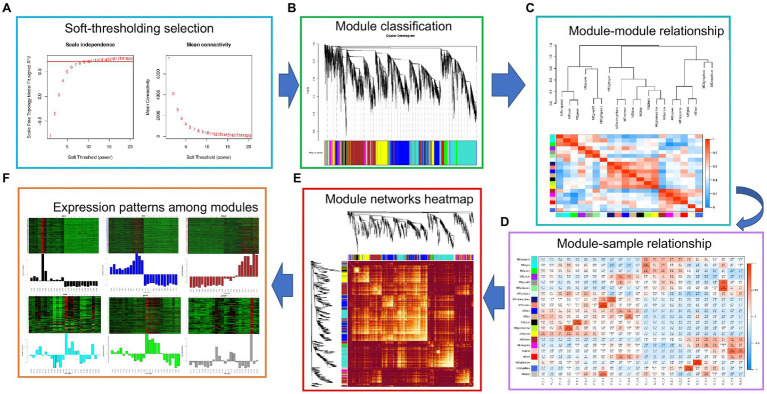
WGCNA analysis. **(A)** Soft threshold selection; **(B)** Module hierarchical clustering tree; **(C)** The gene co-expression module of hickory nut; **(D)** The correlation between modules; **(E)** Cluster analysis of module gene correlation; and **(F)** Gene expression pattern between modules.

WGCNA analysis was performed on 33,268 genes expressed in 24 samples of exocarp and embryo at different development stages according to their expression levels. First, threshold screening was performed according to their expression levels, and the gene expression data network complied with non-scale characteristics (Figure 4A, left). The best *β* (Power Estimate) value is 9 (Figure 4A, right). Secondly, a cluster tree was constructed according to the correlation between gene expression levels. In the cluster tree, each branch in the upper part of the cluster tree represents a module, and the leaf on each branch represents a gene. Each color in the lower part of the module represents each gene in the cluster tree belonging to the same module. As a result, 21 modules were constructed (Figure 4B). The correlation analysis and heat map among 21 modules were subsequently performed (Figures 4C–F).

#### Regulation Network of Core Genes in Flavonoid Biosynthesis in Fruits

According to the statistics of the above 21 modules, it was found that the blue, brown, turquoise, and yellow modules had the most enriched genes, with 3,846, 3,493, 4,958, and 2,395 genes, respectively (Figure 3E). Furthermore, through KEGG functional enrichment analysis of gene sets in these modules, it was found that the blue, brown, turquoise, and yellow modules were related to the flavonoid biosynthesis process in fruit development. The pathways related to flavonoid biosynthesis in the BLUE module were Phenylpropanoid biosynthesis/metabolism, Phenylalanine, tyrosine, tryptophan biosynthesis, and Flavonoid biosynthesis. The brown module is Phenylpropanoid biosynthesis, Flavonoid biosynthesis, Isoflavone biosynthesis, Phenylalanine, tyrosine, and tryptophan biosynthesis. The turquoise module synthesizes Phenylpropanoid biosynthesis/metabolism, Phenylalanine, tyrosine, tryptophan biosynthesis, and Flavonoid biosynthesis. The yellow modules are Phenylpropanoid biosynthesis/metabolism and Flavonoid biosynthesis.

Gene regulatory networks were constructed through the genes enriched in the flavonoid biosynthesis pathway in the four blue, brown, turquoise, and yellow modules to establish the Hub genes of flavonoid biosynthesis in fruit development. The genes with high connectivity probably play a pivotal role in this module (Supplementary Table S4). Therefore, the top 10% of genes in average connectivity were recruited as hub genes. The hub genes related to flavonoid biosynthesis in the BLUE module were *TT4* (*CCA1250S0092*) and *CCOAOMT1* (*CCA0670S0080*). Similarly, the hub genes were *UGT71D1* (*CCA1700S0011*) in BROWN block; *C4H* (*CCA0524S0150*) in TURQUOISE module block; and *F3H* (*CCA0705S0076*), *TT8* (*CCA0510S0065*), *FLS1* (*CCA0533S0160*), and *LDOX* (*CCA1505S0029*) in YELLOW block (Figure 3F). Hence, *TT4*, *CCOAOMT1, UGT71D1, C4H, F3H*, TT8, *FLS1*, and *LDOX* played a key role as hub genes in flavonoid biosynthesis during fruit development.

### Analysis of Flavonoid Metabolites During the Fruit Development

#### Quantitative Analysis of Flavonoid Metabolites

**Figure 5:**
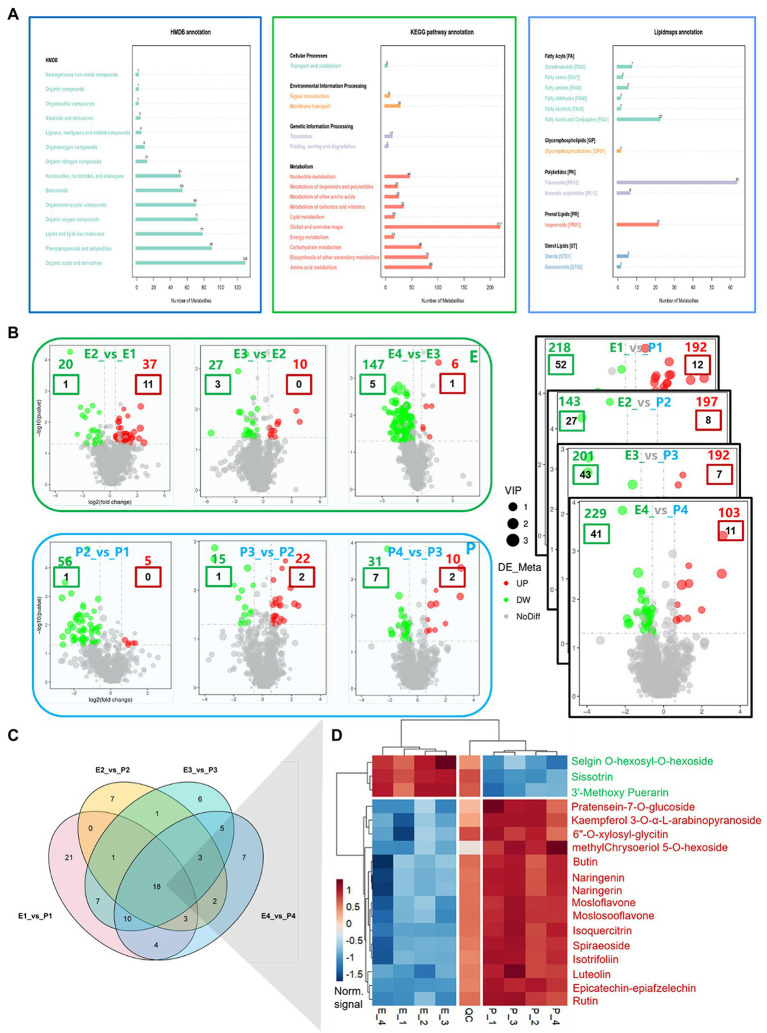
Functional annotation and differential analysis of metabolites, differential expression analysis of flavonoids metabolites between tissues. **(A)** Functional annotations of metabolites; **(B)** Differential analysis of metabolites; **(C)** Venn diagram of DEGs in flavonoid metabolites between tissues; and **(D)** Heat map of DEGs in flavonoid metabolites between tissues. In **(B)**, colored numbers represent the number of differentially expressed metabolites, in which red letters represent upregulated and green letters represent downregulated; the colored boxes represent differentially expressed flavonoid metabolites, with the red boxes representing upregulated and the green boxes representing downregulated.

The correlation (R2) among all QC samples was generally higher than 0.95 (Supplementary Figure S5A), and the principal component analysis showed a high degree of aggregation among all samples (Supplementary Figure S5B), indicating that the samples were highly repeatable and the data quality was reliable. After qualitative and quantitative analysis of metabolites, 1,146 metabolites were obtained. These metabolites can be classified into 57 categories, of which 18 Flavanones, 35 Flavones and Flavonols, 13 Isoflavonoids, and 78 Flavonoids are included. A total of 144 flavonoid-related metabolites were identified (Supplementary Figure S6). Functional annotation of metabolites shows that the metabolites related to flavonoids are mainly concentrated in Phenylpropanoids and polyketides in the HMDB database, and flavonoid biosynthesis in the KEGG database (map00941: Flavonoid biosynthesis), Flavone and flavonol biosynthesis (map00944: Flavone and flavonol biosynthesis), and isoflavone biosynthesis (map00943: Isoflavonoid biosynthesis) and Flavonoids in the Lipidmaps database (Figure 5A).

#### Difference Analysis of Flavonoid Metabolites

With the fruit development, the upregulated metabolites decreased gradually, while the downregulated metabolites increased gradually in the embryo. Upregulated metabolites increased initially and then decreased in the exocarp, while downregulated metabolites gradually decreased (Figure 5B). The expression of metabolites was compared between embryo and exocarp. The differently expressed metabolites between samples were significantly more than those within samples. At the early stage of cotyledon, 192 metabolites (12 flavonoid metabolites) were upregulated, and 218 (52 flavonoid metabolites) were downregulated. In the middle and late cotyledon stages, 197 (8 flavonoids) were upregulated, and 143 (27 flavonoids) were downregulated. A 192 (7 flavonoids) were upregulated at the complete cotyledon stage, and 201 (43 flavonoids) were downregulated. During maturity, 103 (11 flavonoids) were upregulated, and 229 (41 flavonoids) were downregulated. The results showed that the different parts’ metabolic activity was more significant than that in different development stages. The metabolic activity in the exocarp was stronger than that in the embryo. The results of metabolite analysis were consistent with those of transcriptome analysis. Further research revealed that 21, 7, 6, and 7 flavonoid metabolites were time-specific in the early, late, complete, and mature cotyledon stages, respectively (Figures 5C,D). In addition, 18 metabolites were differentially expressed at all developmental stages, including Selgin O-hexosyl-O-hexoside, Sissotrin, 3′-Methoxy Puerarin, Pratensein-7-O-glucoside, Kaempferol 3-O-α-L-arabinopyranoside, 6″-O-xylosyl-glycitin, methylChrysoeriol 5-O-hexoside, Butin, Naringenin, Naringerin, Naringenin 7-Rhamnoglucoside, Mosloflavone, Moslosooflavone, Isoquercitrin, Spiraeoside, Isotrifoliin, Luteolin, Epicatechin-epiafzelechin, and Rutin.

### Analysis of Flavonoid Biosynthesis and Metabolism Pathway During the Development of Hickory Fruit

**Figure 6:**
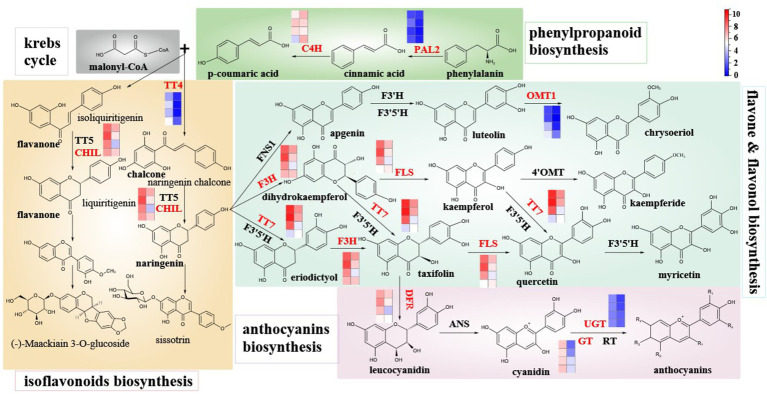
Schematic diagram of main branches of flavonoid biosynthesis in hickory fruit development and expression levels of different enzymes involved in the flavonoid biosynthesis pathway. Note: The FPKM of the above-mentioned differential genes was normalized by Log2, and the average value of 19 genes encoding UGT enzyme was taken. The eight squares of each enzyme were corresponding to exocarp and embryo of hickory fruit at four developmental stages, respectively, 85d, 100d, 109d, and 127d after pollination from top to bottom, with exocarp on the left and embryo on the right.

To further explore the relationship between DEGs and differentially expressed metabolites involved in flavonoid synthesis, this paper systematically constructed the flavonoid biosynthetic pathway during the development of hickory fruit (Figure 6). The pathway includes the upstream flavonoid pathway: phenylpropanoids biosynthesis, Flavones and flavonols biosynthesis, isoflavones biosynthesis, and anthocyanins biosynthesis pathway. Among the 72 DEGs involved in the regulation of flavonoid biosynthesis in fruits, 10 enzymes encoded by 29 DEGs are involved in 4 metabolic pathways of flavonoid biosynthesis. The genes encoding C4H, TT7, F3H, CHIL, TT4, DFR, PAL2, OMT1, GT, FLS, and UGT were found in the mountain nucleus. The enzymes expressed in peach embryos were different in different heat maps. PAL2 catalyzed the transformation of phenylalanine to cinnamic acid, and then to p-coumaric acid under the catalysis of C4H. Malonyl-coa and P-Coumaric acid were used as substrates. TT4 catalyzed the first step of flavonoid biosynthesis. As can be seen from the FPKM value, the expression level of *PAL2* in the embryo was higher than that in the outer exocarp. It gradually decreased as the fruit developed to maturity. The expression level of C4H in the embryo was higher than that in the outer exocarp and decreased with the development of hickory fruit. The expression level of *TT4* in the outer exocarp was significantly higher than that in the embryo and decreased gradually with fruit development to maturity. Upstream substrates catalyzed by TT4 produce isoliquiritigenin and naringeninchalcone, which will initiate two isoflavone biosynthesis pathways. As can be seen from the FPKM value, the *TT4* gene is related to these two isoflavone biosynthesis pathways. Hence, their downstream enzyme CHIL expression level in exocarp is significantly higher than that in the embryo. It indicates that isoflavones are synthesized through these two pathways.

The first pathway is the conversion of Isoliquiritigenin into Liquiritigenin under the collaborative catalysis of TT5 and CHIL, followed by a few steps of catalytic conversion into (-)-Maackiain3-O-glucoside. The other pathway is the conversion of Naringenin chalcone into Naringenin under the cooperative catalysis of TT5 and CHIL and then into Sissotrin after a few steps of catalytic transformation. Naringin, an intermediate in the isoflavone biosynthesis pathway, will initiate the three-step flavonoid and flavonol biosynthesis pathway. The first step is the conversion of Naringenin to Apigenin catalyzed by FNS1, then to Luteolin catalyzed by F3′H and F3′5′H, and then to Chrysoeriol catalyzed by OMT1. According to the FPKM value, the expression level of the *OMT1* gene in exocarp was higher than that in the embryo. The expression level increased gradually with the development of hickory fruit, mainly in the cotyledon completes and fruit maturity stages. In the other step, Naringenin was converted to Dihydrokaempferol catalyzed by F3H, then to Kaempferol catalyzed by FLS, and then to Kaempferide catalyzed by 4′OMT. As can be seen from the FPKM value, the expression levels of F3H and FLS genes in exocarpium were significantly higher than those in the embryo. With fruit development to maturity, the expression levels of F3H and FLS genes gradually decreased, mainly in the middle and late cotyledons. The third step is that Naringenin is transformed into Eriodictyol under F3′5′H and TT7 synergistic catalysis and transformed into Taxifolin after F3H catalysis. Meanwhile, dihydrokaempferol of the second step can also be converted into Taxifolin under the co-catalysis of F3′5′H and TT7 and then into Quercetin under the catalysis of FLS. The second step product kaempferol can also obtain quercetin through F3′5′H and TT7 synergistic catalysis. Quercetin is then converted to Myricetin with the catalysis of F3′5′H. As can be seen from the FPKM value, the expression levels of F3H, TT7, and FLS in exocarpium were significantly higher than those in the embryo. With fruit development to maturity, the expression levels of F3H, TT7, and FLS were gradually decreased, mainly in the middle and late cotyledon. In the third step of the biosynthesis of flavonoids and flavonols, the intermediate taxifolin was converted into leucocyanidin under DFR catalysis, and the anthocyanins biosynthesis pathway was initiated. Leucocyanidin was converted to Cyanidin catalyzed by ANS and then to anthocyanins by enzymes such as UGT, GT, and RT. As seen from the FPKM value, the expression levels of DFR, GT, and UGT in exocarp were significantly higher than those in embryo. Their expression levels tended to decrease with fruit development to maturity. However, among the genes encoding UGT enzyme, some DEGs, such as UGT74E2, UGT91C1, and UGT89B1, were expressed in the embryo relatively higher than in the outer peel. Some DEGs, such as UGT73B1, UGT76E1, and UGT85A4, the expression of these genes in the exocarp was higher than that in the embryo (Supplementary Figure S7).

The above results showed that the expression levels of C4H, UGT74E2, UGT91C1, UGT89B1, and UGT79B3 in the embryo were higher than those in the outer pericardium. Still, the expression levels of these genes decreased gradually with the development of the fruit, mainly in the middle and late cotyledons F3H, PAL2, TT4, TT7, DFR, CHIL, OMT1, GT72B1, FLS1, UGT73B1, UGT76E1, etc. In general, the expression levels of these genes in the outer peel were significantly higher than those in the embryo, and the expression levels decreased gradually as the fruit developed to maturity.

## Discussion

### DEGs Related to Flavonoid Biosynthesis During the Fruit Development

Many studies have shown that flavonoid metabolism in plants is a complex network-regulated process involving many genes. Transcriptomics analysis showed that flavonoid metabolism in pomegranate (Luo et al., 2018), grape (Georgiev et al., 2014), kiwifruit (Hettihewa et al., 2018), and other horticulture plants are closely related to the expression of *PAL*, *CHS*/*TT4*, *4CL*, *FLS*, and *F3H*. In this study, 72 DEGs were directly involved in flavonoid biosynthesis during fruit development. Gene expression in exocarp and embryo could be divided into four categories, Cluster I-IV. Cluster I was involved in synthesizing flavonoids in embryo, and 12 DEGs were found, including *C4H*, *BAN*, *CCoAOMT7*, *UGT74E2*, *UGT89B1*, and *UGT91C1*. Cluster II was involved in synthesizing flavonoids in exocarp, and there were 60 DEGs, including *F3H*, *TT4*, *TT7*, *TT8*, *CCoAOMT1*, *LDOX*, and *Ben1*. In this paper, WGCNA was used to screen the hub genes of flavonoid biosynthesis in fruits. The hub genes involved in flavonoid synthesis in fruits were *TT4* (*CCA1250S0092*)*, CCOAOMT1* (*CCA0670S0080*)*, UGT71D1* (*CCA1700S0011*)*, C4H* (*CCA0524S0150*)*, F3H* (*CCA0705S0076*)*, TT8* (*CCA0510S0065*)*, FLS1* (*CCA0533S0160*), *and LDOX* (*CCA1505S0029*). These genes played an important role in regulating flavonoid biosynthesis and metabolism in hickory.

The formation of polyphenolic compounds is initiated by the phenylpropanoid biosynthetic pathway, known as the starting point for synthesizing a large family of low molecular weight plants secondary metabolites such as lignin, phenolic acids, flavonoids, stilbenes, and lignans (André et al., 2009). In this pathway, some major flavonoids and phenolic acids can be produced by key genes, including phenylalanine-ammonia-lyase (PAL), cinnamate 4 hydroxylase (C4H), chalcone synthase (CHS), Flavanone 3-hydroxylase (F3H), and chalcone isomerase (Ueyama et al., 2002; Mouradov and Spangenberg, 2014).

Jiang et al. identified 53 unigenes encodings 15 enzymes to reveal the flavonoid biosynthesis metabolic pathway in Semen Ziziphi Spinosae. These unigenes were identified as candidate genes involved in flavonoid biosynthesis in SZS. PAL, C4H, 4CL, CHS, CHI, FLS, FNS, ANS, ANR, LAR, and UFGT genes were all downregulated in T3 than in T1. The expression levels of these genes influence the accumulation of flavonoids in SZS (Jiang et al., 2021).

As an intermediate of flavane-3-alcohol biosynthesis in the flavonoid metabolic pathway, F3H can transform flavanones into dihydroflavonols, a key enzyme regulating flavonoid accumulation in plants (Mamati et al., 2006). A large accumulation of F3H transcripts was detected in cultivated species containing high levels of flavonoids (Xiong et al., 2016). Han Yahui et al. cloned two F3H genes, CsF3Ha and CsF3Hb, from tea plants. Uplc-ms/MS was used to analyze the flavonoids in transgenic Arabidopsis seeds and compare them with wild Arabidopsis plants. The results showed that these two genes were in transgenic Arabidopsis seeds. The contents of most flavonol glycosides and oligo-procyanidins increased significantly, while the contents of monomer catechin derivatives decreased. Chalcone synthase (CHS), chalcone isomerase (Ueyama et al., 2002), and flavanone 3-hydroxylase (F3H) have been considered key enzymes in the phenylpropane pathway (Hodaei et al., 2018).

Furthermore, naringenin can be converted to dihydroflavonols, dihydrokaempferol, dihydromyricetin, and dihydroquercetin by flavanone-3-hydroxylase (F3H) activity. Dihydroflavonols can be reduced to respective flavonols by FLS enzyme and finally glycosylated, for example, quercetin to rutin, by flavonoid-3O-glucosyltransferase (UFGT) activity (Ueyama et al., 2002).

Studies have shown that drought enhanced the transcription levels of PAL, C4H, and F3H, genes encoding a key enzyme in enzyme-catalyzed phenylpropanoid biosynthesis, leading to an increase in the flavonoid levels (Gharibi et al., 2019).

### Relationship Between Genes and Metabolites of Different Flavonoid Metabolism in Fruits

Previous studies have found that the content of flavonoids changes rapidly during fruit ripening, and the content of flavonoids decreases when the fruit ripens (Zorenc et al., 2016). In this paper, the changes of flavonoids in the exocarp and embryo were different during the fruit development. Flavonoids content in exocarp generally showed a decreasing trend, consistent with the results of Shi et al. in walnut (*Julgans sigillata* Dode). The possible reason is that in the early stage of fruit development, the young exocarp grows vigorously, the flavonoid metabolism is greater than catabolism, the content of auxin in the vigorous growing organs is higher, and the flavonoid compounds in the storage organs are transported quickly (Shi et al., 2017). In addition, at the fruit ripening stage, the biological process of seed ripening is completed, and the special astringency formed by flavonoid substances in the exocarp is no longer needed to prevent the invasion of insects and pathogens (Persic et al., 2018).

On the contrary, during fruit development, the flavonoid content in the embryo generally increases. Although the membranous endocarp is relatively light in weight, it is the main source of flavonoid substances in the embryo, and its content is very high (Colaric et al., 2005). Therefore, the high content of flavonoids in the embryo is due to the rich content of flavonoids in the membranous endocarp of the embryo.

A total of 144 flavonoids were identified in this paper. The catechin content in exocarp and embryo was higher than that of other flavonoids during the whole fruit development process. In addition to the primary catechins in the exocarp, the high contents were viritin, rutin, and naringin. In contrast, the high contents in the embryo were hydroxymethyl flavonoids O-hexosylhexoside, asanthoside, and 3 ‘-methoxy puerarin. These differences in flavonoid contents may relate to the different metabolic activities in the exocarp and embryo. Plant flavonoids, especially flavonoids, mainly originate from the metabolism of the phenylpropane pathway and are regulated by related enzyme activities (PAL, 4CL, F3H, FLS, etc.; Li et al., 2018). In this study, we found that the gene expression levels of *PAL*, *4CL*, *FLS*, *F3H*, and *F3′5′H* during exocarp development were positively correlated with the contents of catechins, expressed catechins, and myricetin metabolites in flavonoids, indicating that these genes played a positive regulatory role in flavonoid metabolism in Chinese hickory.

### Regulation Pathway of Flavonoid Metabolism in Fruit

This study showed different genes in the flavonoid metabolism pathway in the exocarp and embryo. There were significant differences in the expression of genes related to the flavonoid synthesis of *PAL2*, *C4H*, *TT4*, *TT5*, *TT7, CHIL*, *FLS*, *F3′5′H*, *OMT1*, and *CCOAMT1* in flavonoid metabolism pathways during exocarp development (Figure 6). Furthermore, the expression levels of these genes and their metabolites in the exocarp were significantly higher than those in the embryo.

F3H played a regulatory role in flavonoid biosynthesis in kiwifruit through binding with transcription factors (Forkmann and Martens, 2001). In the study of flavonoid metabolism in Ginkgo biloba, it was found that different genes in the same gene family of *CHS*, *FLS*, *CHI*, *F3′H*, and *DFR* expressed differently under dark and light conditions (Ni et al., 2018). The metabolism of flavonoids in Chinese hickory was regulated by genes such as *C4H*, *BAN*, *CCoAOMT7*, *F3H, TT4*, *TT7*, *TT8*, *UGT71D1*, *CCoAOMT1*, *LDOX*, and *Ben1*, transcription factors such as *MYB11*, *MYB12*, and *MYBD*, and environmental factors. *TT4*, *CCoAOMT1*, *UGT71D1*, *C4H*, *F3H*, *TT8*, *FLS1*, and *LDOX* are the hub genes of flavonoid metabolism in fruits.

We found that during the development of hickory fruit, 10 enzymes encoded by 29 DEGs were involved in 4 metabolic pathways of flavonoid biosynthesis. All genes encoding 10 enzymes were expressed in hickory embryos. This study clarified the expression of DEGs in the flavonoid biosynthesis pathway during the development of hickory fruit, which will provide a strong research basis for further exploration of flavonoid metabolism in hickory.

## Conclusion

The main conclusions are as follows: A total of 72 DEGs were involved in regulating flavonoid biosynthesis during the development of hickory fruit. There were 12 DEGs involved in embryo flavonoid biosynthesis, including C4H, BAN, and CCoAOMT7. There were 60 DEGs involved in flavonoid biosynthesis in exocarp, including F3H, TT4/7/8, CCoAOMT1, LDOX, and Ben1. TT4, CCoAOMT1, UGT71D1, C4H, F3H, TT8, FLS1, and LDOX are the core genes involved in synthesizing fruit flavonoids.

The changes of flavonoids in exocarp and embryo were different during fruit development. The content of flavonoids in exocarp showed a decreasing trend, but the change in the embryo was the opposite. In addition, the expression levels of hydroxymethyl flavone O-hexosylhexoside, dauberosin, and 3′-methoxy puerarin were higher in the embryos. However, purple rivet, rutin, and naringin were highly expressed in the outer exocarp. Differential genes in flavonoid metabolism during exocarp and embryo development play a regulatory role in differential metabolites in exocarp and embryo, respectively, but the number and expression trend of differential genes and differential metabolites in exocarp and embryo is different.

A 1,146 metabolites were detected by metabolite analysis, including 144 flavonoids-related metabolites. The analysis of differential metabolites showed that at the same developmental stage, the downregulated metabolites were more than the upregulated metabolites in the embryo than in the exocarp; in different development stages of the same part, with the fruit development, the downregulated metabolites gradually increased, while the upregulated metabolites gradually decreased, which indicates that the difference of hickory metabolite activity in different parts is higher than that in different development stages, and the metabolic activity in exocarp is stronger than that in embryo, which is highly consistent with the results of transcriptome analysis.

The different genes involved in the biological metabolism of flavonoids in the pericarp and embryo of hickory may be caused by the changes of the metabolic law of flavonoids in the pericarp and embryo, the differences of flavonoids species and development sites. In addition, the expression abundance and regularity of genes involved in flavonoid biosynthesis in hickory pericarp and embryo are also different. The possible reason is that the regulation of flavonoid metabolism is not only affected by relevant genes, but also significantly regulated by transcription factors, such as *MYB11*, *MYB12*, and *MYBD* found in this paper and the external environment. The flavonoid metabolism in hickory fruit starts from the phenylpropane pathway and then moves to the biosynthesis of ketone and flavonol, the biosynthesis of isoflavone, and the biosynthesis anthocyanin. The results confirmed that the DEGs play a regulatory role in the metabolism of flavonoids.

Hickory kernel is rich in flavonoids, but its flavonoid biosynthesis and metabolism are not clear. Therefore, through the combination of transcriptome and metabolome, this paper studies the metabolism of flavonoids in hickory fruits at different development stages, which is expected to preliminarily reveal the related genes and metabolic pathways of hickory flavonoids metabolism at the “omics” level, in order to lay a foundation for the study of plant flavonoids.

## Data Availability Statement

The datasets presented in this study can be found in online repositories. The names of the repository/repositories and accession number(s) can be found at National Center for Biotechnology Information (NCBI) BioProject database under accession number PRJNA793525.

## Author Contributions

J-HC: visualization, methodology, formal analysis, data curation, conceptualization, writing—original draft, and writing—review and editing. NH: conceptualization, methodology, and writing—review and editing. XX: conceptualization, methodology, investigation resource, data curation, and writing—review and editing. DZ: data curation, visualization, methodology, and writing—review and editing. T-QF: data curation, visualization, and methodology. Q-XZ: writing—review and editing. Y-JH: conceptualization, supervision, writing—review and editing, project administration, and funding acquisition. All authors contributed to the article and approved the submitted version.

## Funding

The study was financially supported by the National Natural Science Foundation of China (grant numbers 31971672, 32171815, and 31860215); the Key Research and Development Project of Zhejiang Province, China (no. 2020C02005); the Natural Science Foundation of Zhejiang Province (LY18C150002); the Zhejiang Key Research and Development project 2021C02037; the Bridging Project for the Enterprises and the Local walnut R&D Groups in Guizhou Province (grant number [2015] 4010); and the Guizhou Kehe platform talent [2019]5643.

## Conflict of Interest

The authors declare that the research was conducted in the absence of any commercial or financial relationships that could be construed as a potential conflict of interest.

## Publisher’s Note

All claims expressed in this article are solely those of the authors and do not necessarily represent those of their affiliated organizations, or those of the publisher, the editors and the reviewers. Any product that may be evaluated in this article, or claim that may be made by its manufacturer, is not guaranteed or endorsed by the publisher.

## Supplementary Material

The Supplementary Material for this article can be found online at: https://www.frontiersin.org/articles/10.3389/fpls.2022.896421/full#supplementary-material

## Footnotes

^1^ http://gigadb.org/dataset/100571

^2^ http://www.broadinstitute.org/gsea/index.jsp
