# Supplementary material for: Flavonoid Synthesis and Metabolism During the Fruit Development in Hickory (Carya cathayensis)
Source: Front Plant Sci. 2022 May 9;13:896421. doi: 10.3389/fpls.2022.896421 (PMC9125235; doi:10.3389/fpls.2022.896421)
Supplement: Supplementary file 1 [file Data_Sheet_1.docx]

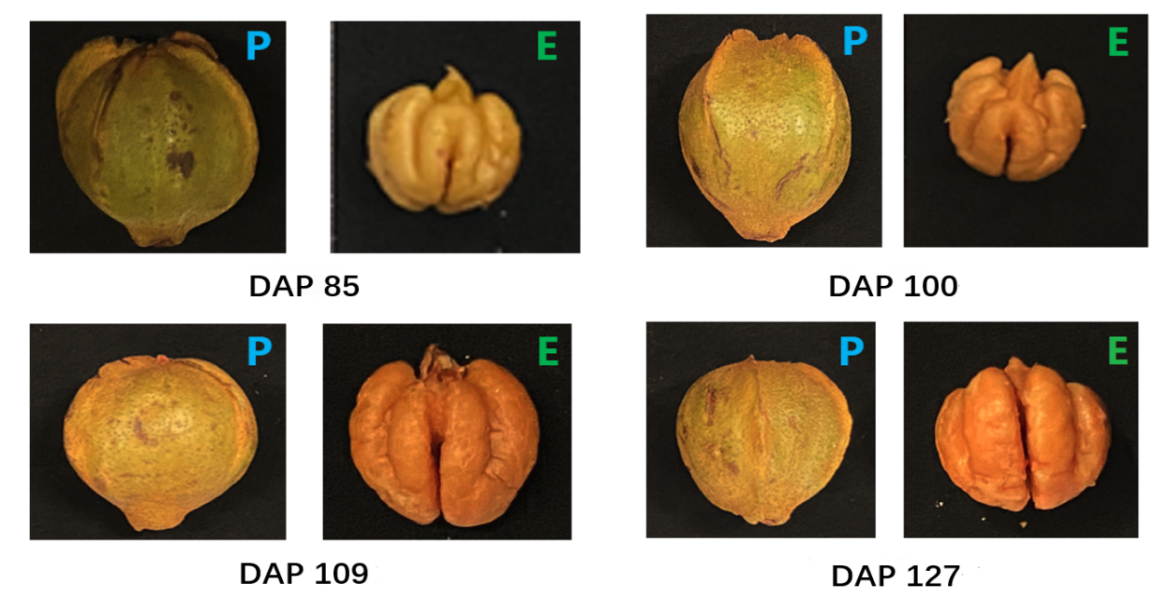


**Figure S1 Appearance traits in different developmental stages of hickory fruits**

Note: DAP, day after pollination: P, epicarp; E: embryo


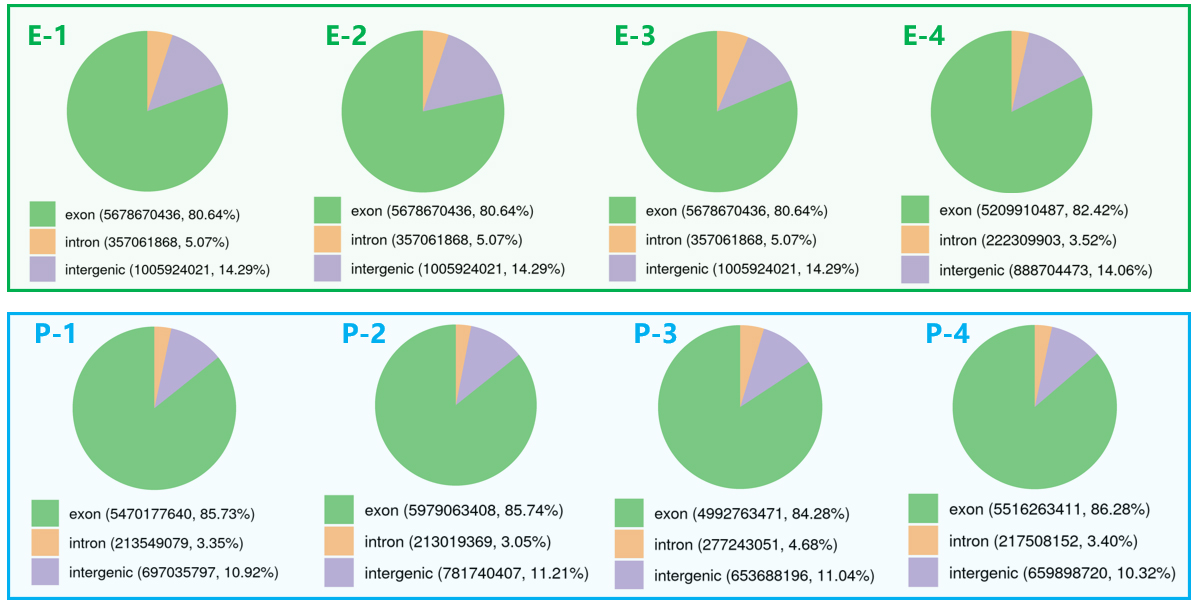


**Figure S2 Reads coverage across different gene regions**


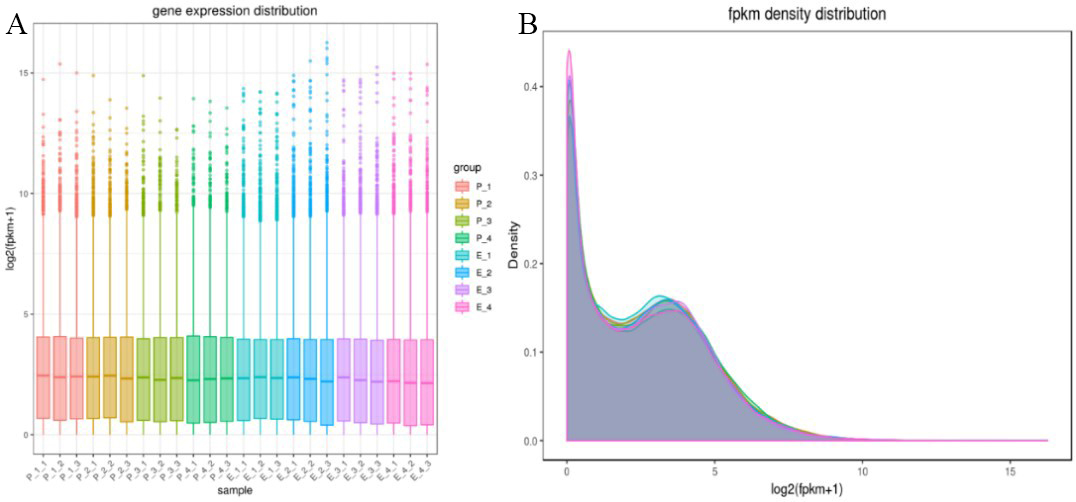


**Figure S3 Gene abundance distribution**


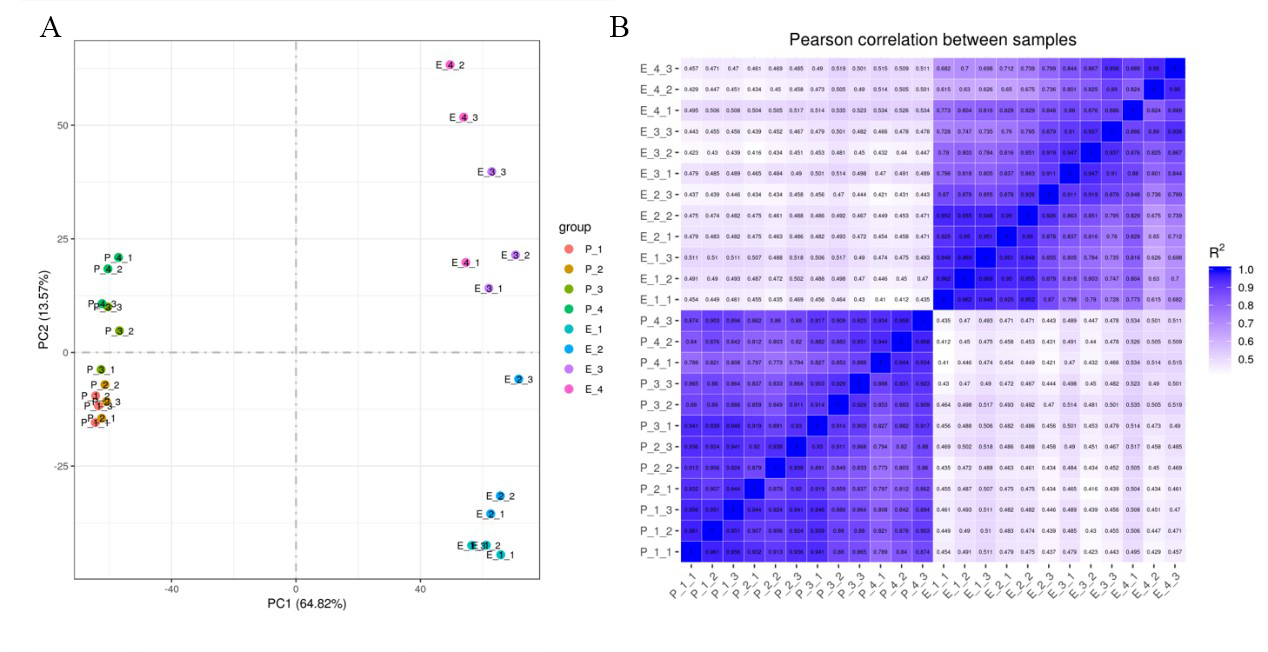


**Figure S4 Correlation analysis between samples**

A. Principal component analysis between samples; B. Inter-sample correlation heat map.


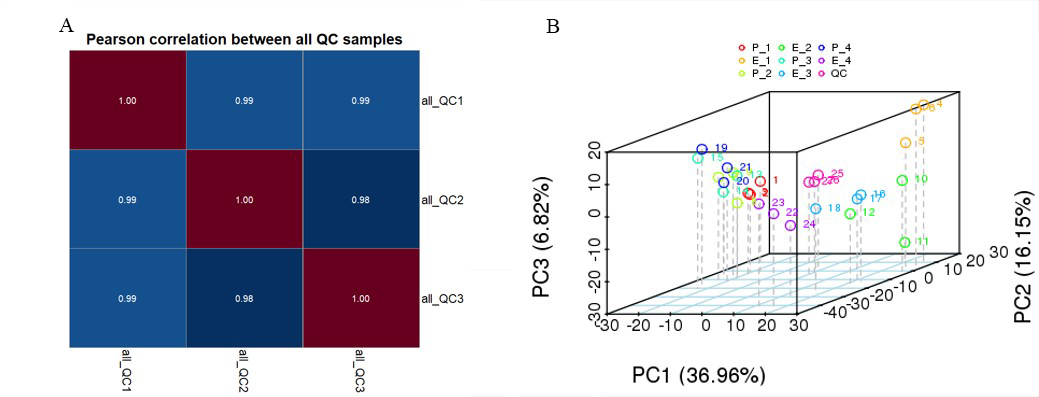


**Figure S5 Sample QCs of hickory metabolites**

A. Pearson correlation between all QC samples; B. Inter-sample principal component analysis


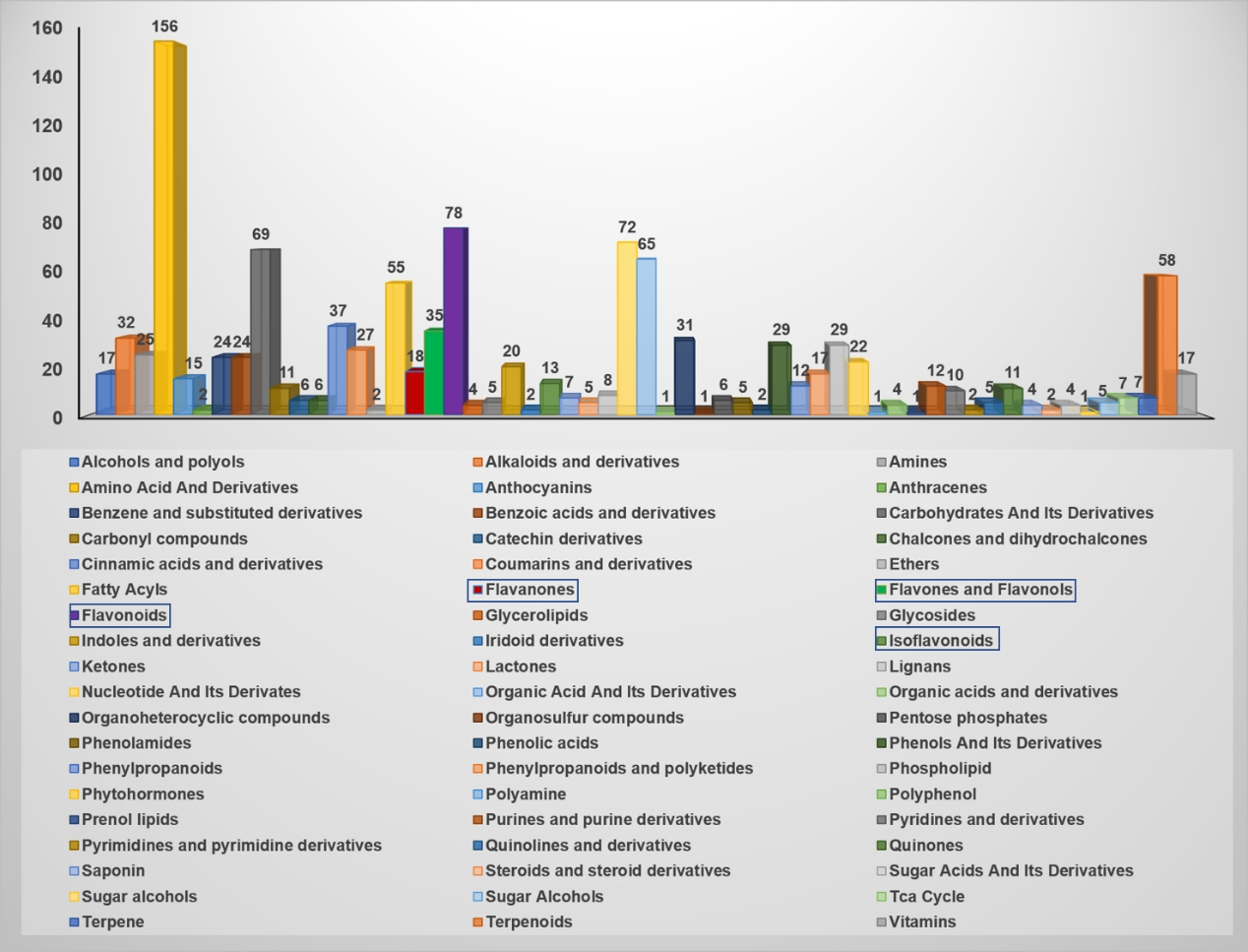


**Figure S6 Classifications of metabolites**

Note: The metabolites in the blue box are those involved in the flavonoid metabolic pathway

**Figure S7 Expression levels of 19 genes encoding UGT enzymes**

The FPKM values of the above-mentioned differential genes encoding UGT enzyme were normalized by Log2. The eight squares of each enzyme were corresponding to the exocarp and embryo of the fruit at 85d, 100d, 109d and 127d after pollination, respectively, from top to bottom, with exocarp on the left and embryo on the right.

**Table S1 Flavonoid biosynthetic related genes during hickory fruit development**

| Abbreviations | English name |
| --- | --- |
| CYP75B1 | Flavonoid 3'-monooxygenase |
| UGT71D1 | Flavonol 3-O-glucosyltransferase |
| UGT88A1 | UDP-Glucosyl Transferase 88A1 |
| UGT76C2 | Cytokinin-N-glucosyltransferase 2 |
| BEN1 | dihydroflavonol 4-reductase （DFR）-like |
| UGT74B1 | Thiohydroximate S-glucosyltransferase |
| MYB12 | Transcription factor MYB12 |
| UGT73D1 | UDP-glycosyltransferase 73D1 |
| UGT85A4 | UDP-glycosyltransferase 85A4 |
| TT8 | Transcription factor TT8 |
| BAN | Anthocyanidin reductase |
| F3H | Flavanone 3-hydroxylase |
| UGT74F2 | UDP-glycosyltransferase 74F2 |
| GT72B1 | UDP-Glycosyltransferase superfamily protein |
| IAGLU | UDP-glycosyltransferase 75D1 |
| UGT84A3 | Hydroxycinnamate glucosyltransferase 3 |
| UGT89A2 | UDP-glycosyltransferase 89A2 |
| CHIL | chalcone--flavonone isomerase 3 |
| UGT76E1 | UDP-glycosyltransferase 76E1 |
| UGT71C4 | Flavonol 3-O-glucosyltransferase UGT71C4 |
| FLS1 | Flavonol synthase/flavanone 3-hydroxylase |
| TT4 | Chalcone synthase |
| UGT73B1 | UDP-glycosyltransferase 73B1 |
| CCoAOMT1 | Caffeoyl-CoA O-methyltransferase 1 |
| LDOX | Leucoanthocyanidin dioxygenase |
| UGT79B3 | UDP-glycosyltransferase 79B3 |
| UGT89B1 | Flavonol 3-O-glucosyltransferase UGT89B1 |
| C4H | Trans-cinnamate 4-monooxygenase |
| PAL2 | Phenylalanine ammonia-lyase 2 |
| 4CLL1 | 4-coumarate--CoA ligase-like 1 |
| MYB11 | Transcription factor MYB11 |
| OMT1 | Flavone 3'-O-methyltransferase 1 |
| MYBD | MYB-like Domain transcription factor |
| At5g66230 | Chalcone-flavonone isomerase family protein |
| UGT91A1 | UDP-glycosyltransferase 91A1 |
| UGT73C3 | UDP-glycosyltransferase 73C3 |
| UGT91C1 | UDP-glycosyltransferase 91C1 |

**Table S2 Statistical results of comparison of reference sample with reference genomees**

| sample | raw_reads | clean_reads | total_map | unique_map | multi_map |
| --- | --- | --- | --- | --- | --- |
| E_1_1 | 50781824 | 48867928 | 47072351（96.33%） | 45547857（93.21%） | 1524494（3.12%） |
| E_1_2 | 47888050 | 46274998 | 44624190（96.43%） | 43138200（93.22%） | 1485990（3.21%） |
| E_1_3 | 45407996 | 43750984 | 42234313（96.53%） | 40844058（93.36%） | 1390255（3.18%） |
| E_2_1 | 41772210 | 39534736 | 38075769（96.31%） | 36394290（92.06%） | 1681479（4.25%） |
| E_2_2 | 46997724 | 45456468 | 43930365（96.64%） | 42597191（93.71%） | 1333174（2.93%） |
| E_2_3 | 41390914 | 39985708 | 38778045（96.98%） | 37555609（93.92%） | 1222436（3.06%） |
| E_3_1 | 41557318 | 39996194 | 38597700  （96.5%） | 37345673（93.37%） | 1252027（3.13%） |
| E_3_2 | 47783774 | 46053902 | 44477277（96.58%） | 43020995（93.41%） | 1456282（3.16%） |
| E_3_3 | 46680478 | 44316688 | 42851048（96.69%） | 41481952  （93.6%） | 1369096（3.09%） |
| E_4_1 | 46049060 | 44268996 | 42257432（95.46%） | 40923413（92.44%） | 1334019（3.01%） |
| E_4_2 | 41937548 | 40537402 | 38949560（96.08%） | 37557527（92.65%） | 1392033（3.43%） |
| E_4_3 | 46797184 | 45095422 | 43529601（96.53%） | 42049650（93.25%） | 1479951（3.28%） |
| P_1_1 | 45985356 | 44356614 | 42641224（96.13%） | 41045298（92.53%） | 1595926  （3.6%） |
| P_1_2 | 45146406 | 43565652 | 42146279（96.74%） | 40631354（93.26%） | 1514925（3.48%） |
| P_1_3 | 47707062 | 45907730 | 44253539  （96.4%） | 42543605（92.67%） | 1709934（3.72%） |
| P_2_1 | 50548918 | 48724968 | 46606838（95.65%） | 44768634（91.88%） | 1838204（3.77%） |
| P_2_2 | 46353030 | 44699144 | 42731128  （95.6%） | 41084681（91.91%） | 1646447（3.68%） |
| P_2_3 | 47441446 | 46201146 | 44868767（97.12%） | 43123592（93.34%） | 1745175（3.78%） |
| P_3_1 | 42539252 | 41160540 | 39585886（96.17%） | 38113026  （92.6%） | 1472860（3.58%） |
| P_3_2 | 46771314 | 45533942 | 44323259（97.34%） | 42748041（93.88%） | 1575218（3.46%） |
| P_3_3 | 46269610 | 44817228 | 43489924（97.04%） | 41937867（93.58%） | 1552057（3.46%） |
| P_4_1 | 45720768 | 44295596 | 42722127（96.45%） | 41127168（92.85%） | 1594959  （3.6%） |
| P_4_2 | 48845932 | 47152330 | 45991243（97.54%） | 44369119  （94.1%） | 1622124（3.44%） |
| P_4_3 | 45935194 | 44870288 | 43540793（97.04%） | 41924079（93.43%） | 1616714  （3.6%） |

**Table S3 Classifications and functions of differentially expressed genes that involved in flavonoid biosynthesis**

| **Cluster** | **hickory ID** | **arab. ID** | **symbol** | **Enzyme** |
| --- | --- | --- | --- | --- |
| I | CCA1106S0015 | AT1G75780 | TUB1 | Tubulin beta-1 chain |
| I | CCA0784S0137 | AT5G23860 | TUB8 | Tubulin beta-8 chain |
| I | CCA0505S0100 | AT4G14960 | TUA6 | Tubulin beta-6 chain |
| I | CCA1057S0060 | AT4G26220 | CCoAOMT7 | Cation-dependent O-methyltransferase 7 |
| I | CCA1209S0008 | AT1G08450 | CRT3 | Calreticulin-3 |
| I | CCA0822S0057 | AT1G61720 | BAN | Anthocyanidin reductase |
| I | CCA0929S0024 | AT1G05680 | UGT74E2 | UDP-glycosyltransferase 74E2 |
| I | CCA0836S0020 | AT5G49690 | UGT91C1 | UDP-glycosyltransferase 91C1 |
| I | CCA0591S0126 | AT1G73880 | UGT89B1 | Flavonol 3-O-glucosyltransferase UGT89B1 |
| I | CCA0539S0265 | AT3G14530 | GFPPS1 | UDP-glycosyltransferase 91C1 |
| I | CCA0524S0150 | AT2G30490 | C4H | Trans-cinnamate 4-monooxygenase |
| I | CCA0539S0062 | AT2G34250 | AT2G34250 |  |
| II | CCA1034S0038 | AT1G04820 | TUA4 | Tubulin alpha-4 chain |
| II | CCA1620S0012 | AT5G42800 | DFR | Dihydroflavonol 4-reductase |
| II | CCA0573S0147 | AT1G75280 | AT1G75280 | Isoflavone reductase homolog P3 |
| II | CCA0800S0145 | AT4G20890 | TUB9 | Tubulin beta-9 chain |
| II | CCA1294S0010 | AT2G19070 | SHT | Spermidine hydroxycinnamoyl transferase |
| II | CCA0623S0133 | AT5G12250 | TUB6 | Tubulin beta-6 chain |
| II | CCA0678S0067 | AT4G39230 | PCBER1 | Phenylcoumaran benzylic ether reductase 1 |
| II | CCA0795S0041 | AT5G07990 | TT7 | Flavonoid 3'-monooxygenase |
| II | CCA1700S0011 | AT2G29730 | UGT71D1 | Flavonol 3-O-glucosyltransferase UGT71D1 |
| II | CCA0559S0124 | AT3G16520 | UGT88A1 | UDP-glycosyltransferase 88A1 |
| II | CCA0884S0183 | AT5G05860 | UGT76C2 | UDP-glycosyltransferase 76C2 |
| II | CCA0638S0167 | AT1G49390 | AT1G49390 | 2-oxoglutarate （2OG） and Fe（II）-  dependent oxygenase superfamily protein |
| II | CCA0546S0102 | AT2G45400 | BEN1 | dihydroflavonol 4-reductase （DFR）-like |
| II | CCA0890S0054 | AT1G24100 | UGT74B1 | Thiohydroximate S-glucosyltransferase |
| II | CCA0953S0011 | AT2G47460 | MYB12 | Transcription factor MYB12 |
| II | CCA1151S0045 | AT3G53150 | UGT73D1 | UDP-glycosyltransferase 73D1 |
| II | CCA1015S0108 | AT5G66230 | AT5G66230 | Chalcone-flavonone isomerase family protein |
| II | CCA0646S0149 | AT1G78270 | UGT85A4 | UDP-glycosyltransferase 85A4 |
| II | CCA0510S0065 | AT4G09820 | TT8 | Transcription factor TT8 |
| II | CCA0705S0076 | AT3G51240 | F3H | Flavanone 3-hydroxylase |
| II | CCA0941S0058 | AT2G43820 | UGT74F2 | UDP-glycosyltransferase 74F2 |
| II | CCA0736S0021 | AT4G01070 | GT72B1 | UDP-Glycosyltransferase superfamily protein |
| II | CCA1327S0002 | AT4G15550 | IAGLU | UDP-glycosyltransferase 75D1 |
| II | CCA0685S0001 | AT4G15490 | UGT84A3 | Hydroxycinnamate glucosyltransferase 3 |
| II | CCA1758S0015 | AT5G03490 | UGT89A2 | UDP-glycosyltransferase 89A2 |
| II | CCA1503S0010 | AT5G05270 | CHIL | chalcone--flavonone isomerase 3 |
| II | CCA0749S0044 | AT2G36970 | AT2G36970 | Glycosyltransferase |
| II | CCA0680S0121 | AT5G59580 | UGT76E1 | UDP-glycosyltransferase 76E1 |
| II | CCA0879S0011 | AT4G25300 | AT4G25300 | 2-oxoglutarate （2OG） and Fe（II）-dependent oxygenase superfamily protein |
| II | CCA1805S0002 | AT1G07250 | UGT71C4 | Flavonol 3-O-glucosyltransferase UGT71C4 |
| II | CCA0533S0160 | AT5G08640 | FLS1 | Flavonol synthase/flavanone 3-hydroxylase |
| II | CCA1250S0092 | AT5G13930 | TT4 | Chalcone synthase |
| II | CCA1094S0055 | AT2G16890 | AT2G16890 | Glycosyltransferase |
| II | CCA0881S0032 | AT2G36780 | AT2G36780 | Glycosyltransferase |
| II | CCA1326S0044 | AT4G34138 | UGT73B1 | UDP-glycosyltransferase 73B1 |
| II | CCA0844S0032 | AT3G21560 | UGT84A2 | UDP-glycosyltransferase 84A2 |
| II | CCA0670S0080 | AT4G34050 | CCoAOMT1 | Caffeoyl-CoA O-methyltransferase 1 |
| II | CCA1505S0029 | AT4G22880 | LDOX | Leucoanthocyanidin dioxygenase |
| II | CCA0925S0030 | AT4G27570 | UGT79B3 | UDP-glycosyltransferase 79B3 |
| II | CCA0662S0078 | AT1G17020 | SRG1 | Protein SRG1 |
| II | CCA0431S0010 | AT5G66690 | UGT72E2 | Hydroxycinnamate 4-beta-glucosyltransferase UGT72E2 |
| II | CCA1543S0017 | AT1G22360 | UGT85A2 | UDP-glycosyltransferase 85A2 |
| II | CCA1564S0010 | AT2G25180 | RR12 | 30S ribosomal protein S12 |
| II | CCA0576S0103 | AT4G27430 | CIP7 | COP1-interacting protein 7 |
| II | CCA0589S0228 | AT3G53260 | PAL2 | Phenylalanine ammonia-lyase 2 |
| II | CCA1287S0042 | AT1G62940 | 4CLL1 | 4-coumarate--CoA ligase-like 1 |
| II | CCA1126S0091 | AT2G02990 | RNS1 | Ribonuclease 1 |
| II | CCA0526S0212 | AT2G43000 | JUB1 | Transcription factor JUNGBRUNNEN 1 |
| II | CCA0633S0009 | AT3G62610 | MYB11 | Transcription factor MYB11 |
| II | CCA0918S0075 | AT5G54160 | OMT1 | Flavone 3'-O-methyltransferase 1 |
| II | CCA0507S0279 | AT1G70000 | MYBD | MYB-like Domain transcription factor |
| II | CCA0633S0099 | AT1G09530 | PIF3 | Transcription factor PIF3 |
| II | CCA1017S0007 | AT2G20570 | GLK1 | Transcription activator GLK1 |
| II | CCA1769S0023 | AT4G15560 | DXS | 1-deoxy-D-xylulose-5-phosphate synthase |
| II | CCA0659S0213 | AT4G34740 | ASE2 | Amidophosphoribosyltransferase 2 |
| II | CCA0862S0116 | AT5G13630 | CHLH | Magnesium-chelatase subunit ChlH |
| II | CCA0975S0060 | AT5G18660 | DVR | Divinyl chlorophyllide a 8-vinyl-reductase |
| II | CCA0737S0008 | AT1G27450 | APT1 | Adenine phosphoribosyltransferase 1 |
| II | CCA0718S0099 | AT1G58290 | HEMA1 | Glutamyl-tRNA reductase 1 |
| II | CCA0500S0074 | AT5G43860 | CLH2 | Chlorophyllase-2 |

**Table S4 GeneSet in four modules that participate in flavonoid biosynthesis**

| **blue** | **brown** | **turquoise** | **yellow** |
| --- | --- | --- | --- |
| CCA1034S0038 | CCA0925S0030 | CCA0638S0167 | CCA0795S0041 |
| CCA0680S0121 | CCA1543S0017 | CCA1106S0015 | CCA0705S0076 |
| CCA1738S0001 | CCA0836S0020 | CCA0784S0137 | CCA1503S0010 |
| CCA0749S0044 | CCA0929S0024 | CCA0505S0100 | CCA0533S0160 |
| CCA1151S0045 | CCA0591S0126 | CCA0881S0032 | CCA1015S0108 |
| CCA1805S0002 | CCA1700S0011 | CCA0524S0150 | CCA0507S0279 |
| CCA0670S0080 | CCA0662S0078 | CCA1057S0060 | CCA1620S0012 |
| CCA1250S0092 | CCA1564S0010 | CCA0539S0062 | CCA0633S0009 |
| CCA0844S0032 |  | CCA0736S0021 | CCA0737S0008 |
| CCA1327S0002 |  | CCA0633S0099 | CCA0646S0149 |
| CCA0918S0075 |  |  | CCA0573S0147 |
| CCA0862S0116 |  |  | CCA0510S0065 |
| CCA0526S0212 |  |  | CCA0800S0145 |
| CCA0879S0011 |  |  | CCA1294S0010 |
| CCA0546S0102 |  |  | CCA0623S0133 |
| CCA1126S0091 |  |  | CCA0576S0103 |
| CCA1769S0023 |  |  | CCA1505S0029 |
| CCA0685S0001 |  |  | CCA0890S0054 |
| CCA0559S0124 |  |  | CCA1758S0015 |
| CCA0941S0058 |  |  | CCA0975S0060 |
|  |  |  | CCA0500S0074 |
|  |  |  | CCA0953S0011 |
